# Painful Pregnancies and Sub-Involution

**Published:** 1893-09

**Authors:** 


PAINFUL PREGNANCIES AND SUB-INVOLUTION; THEIR PREVENTION, by Wm. F. Kier, M. D., St. Louis, Mo.—The busy practitioner is constantly impressed with the fact that the habit of life of the average woman in the direction of constipation, improper clothing, tight lacing, over-strained nerves, and undeveloped, flabby muscular system, tends toward pelvic congestion and plastic exudations in the reproductive organs, inducing a class of lesions, such as metritis, metrorrhagia, ovarian neuralgia, pelvic cellulitis, as well as being the direct cause of painful pregnancies, after-pains and sub-involution.

Have we had a handy remedy, easy of administration, which will assist us in anticipating and preventing these evils? According to my experience we have. Do you ask me what it is? It is "Ponca Compound" furnished to us in tablet form, each tablet containing: Extract Ponca, 3 grains; Extract Mitchella Repens, 1 grain; Caulophyllin one-fourth of a grain; Helonin, one-third of a grain; Virburnin, one-eighth of a grain.

It commended itself to me some years since, and each year's success has confirmed the correctness of my early conclusions so that after extensive clinical experience I am convinced that it exercises a specific alterative action on the uterine tissues, a general tonic influence on the pelvic organs; that it has a tendency to absorb plastic deposits, to regulate the vascular supply to relieve congestion, to encourage peristalsis of the bowels and to remove spasmodic conditions.

In fact we have in the Ponca Compound that which is uniform, portable, convenient, reliable and agreeable, indeed—"a definite chemical compound." I confidently commend it to my co-workers of the medical profession.

T. H. J. PRYCE, M. D., etc., No. 4 Lorne Villas, Clevedon, Somerset, England, May 23d, 1891, writes:

I take pleasure in giving the following notes on BROMIDIA. A patient, age 28, suffering from Pneumonia and Typhoid Blood Poisoning (the latter was contracted when in the convalescent stage), complained of Insomnia, and I put him on BROMIDIA. Even when in good health he had suffered more or less from Insomnia, but after having taken BROMIDIA he slept without difficulty and very naturally, and no headache or constipation followed its use, as was the case when other narcotics were administered. I was very pleased with the results, and prescribe BROMIDIA often now.

---

I use Anticlavus in preference to all other antipyretics.

Clayton, Ill., Oct. 30, '92. J. P. HARREL, M. D.

